# Extracorporeal Membrane Oxygenation for Complex Multiorgan System Trauma

**DOI:** 10.1155/2012/897184

**Published:** 2012-03-29

**Authors:** Michael S. Firstenberg, Karen Nelson, Erik Abel, John McGregor, Daniel Eiferman

**Affiliations:** ^1^Division of Cardiac Surgery, The Ohio State University Wexner Medical Center, Columbus, OH 43210, USA; ^2^Department of Neurosurgery, The Ohio State University Wexner Medical Center, Columbus, OH 43210, USA; ^3^Division of Trauma and Critical Care Surgery, The Ohio State University Wexner Medical Center, Columbus, OH 43210, USA

## Abstract

With growing experience, the indications for salvage extracorporeal membrane oxygenation continue to expand. We describe a successful application of extracorporeal support in a polytrauma patient presenting with profound hypothermia, respiratory failure, and whom was later found to have an intracranial hemorrhage. We advocate the role of salvage therapy even in patients with complex pathophysiology despite perceived relative or absolute contraindications to extracorporeal support.

## 1. Introduction

The use of extracorporeal membrane oxygenation (ECMO) has been shown to be an acceptable intervention for patients with respiratory failure refractory to optimal ventilator management [1]. As experience with ECMO grows the indications for use also broaden. For example, patients with profound hypothermia have been also shown to benefit from central controlled rewarming with ECMO [2]. Furthermore, while ECMO has been successfully described in patients with trauma-induced respiratory failure, these patients have typically undergone extensive evaluation and primary management of their injuries. In these patients, ECMO is typically initiated several days after admission and after a traumatic brain injury has been ruled out in part because ECMO has traditionally been contraindicated in those with central nervous system injuries [3]. We present the successful early application of ECMO in a patient who presented with profound hypothermia, acute respiratory failure, and an intraventricular hemorrhage following a motor vehicle accident.

## 2. Case

**Figure 1:**
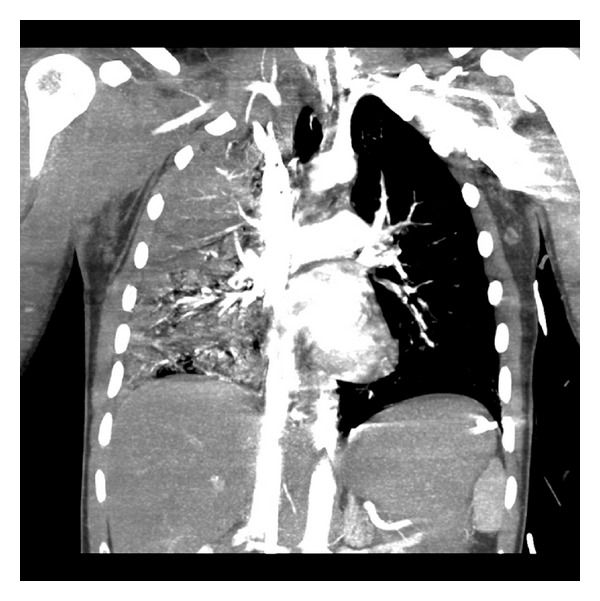
Computed tomography (CT) of the chest demonstrates extent of acute pulmonary injury.

**Figure 2:**
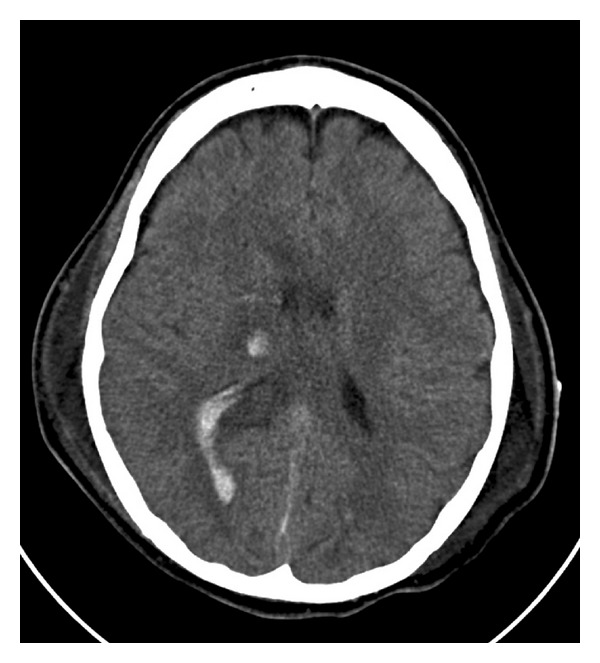
CT of head demonstrates intraventricular and intraparenchymal blood.

Our patient is a 27-year-old male who was found unresponsive after a motor vehicle accident. He was intubated at the scene and transferred to our institution. Upon arrival his heart rate (71 bpm) and blood pressure were stable (139/97 mmHg). However, he was hypothermic so we were unable to obtain a peripheral temperature with a combined respiratory and metabolic acidosis (pH = 7.23, pCO_2 _= 63 mmHg, pO_2_ = 62 mmHg on 100% oxygen) and despite topical warming and mechanical ventilation, he remained hypothermic (<33C) with worsening acidosis and hypoxemia (pH = 7.19, pCO_2_ = 49 mmHg, pO_2_ = 56 mmHg, base excess −10, saturating 82% on assist control, rate = 22, FI02 = 1.0, PEEP = 15 mmHg, and 20,000 ng/mL of inhaled epoprostenol sodium). It was felt that he would potentially benefit from salvage ECMO. Following systemic heparinization (10,000 Units), percutaneous femoral-femoral veno-veno ECMO was initiated with a 23F inflow cannula positioned in the inferior vena cava and a 21F cannula at the level of the right atrium using a Medtronic Biomedicus BP-80 pump and tubing circuit (Minneapolis, MN, USA) and a Maquet Quadrox D oxygenator (Wayne, NJ, USA). Initial flows were 4.2 liters/min on 100% oxygen. He was actively warmed to normothermia over 8 hours. Once stable, he was evaluated for other major injuries. A CT of the chest suggested a pulmonary contusion with consolidation consistent with an acute aspiration (Figure 1). A CT scan of the head showed multiple foci of intraparenchymal hemorrhage with intraventricular extension, subdural blood, right-sided edema, and midline shift (Figure 2). Due to his unresponsiveness and CT scan findings, a cerebral intraventricular pressure monitor was placed. A transthoracic echocardiogram was unremarkable. Bronchoscopy failed to show any obvious obstruction, just diffuse airway edema. Repeat head CT on posttrauma days 1 and 5 showed no interval changes. Forty-eight hours after cannulation, heparin was started and maintained at 4.0 units/kg/hour. Evaluation of the ECMO circuit every 6–8 hours, despite the absence of anticoagulation initially, did not show any evidence of thrombus formation or change in gas exchange efficiency (with post circuit blood gases), and a single oxygenator was used for the entire duration of therapy. Approximately 96 hours after cannulation, he was taken to the operating room for ECMO decannulation. Prior to leaving the operating room on assist control (FI0_2_ = 1.0, PEEP = 12, rate 15), his arterial blood gas was pH = 7.48, pCO_2_= 35 mmHg, pO_2_ = 296 mmHg, and base excess −1.5. Several days later, he underwent tracheostomy and feeding tube placement. His intraventricular monitor was subsequently removed.

On posttrauma day 23, he was transferred to an inpatient rehabilitation facility. At the time of discharge, he was saturating >95% on room air, grossly neurologically intact although he had evidence of short-term memory impairment and diffuse deconditioning. Twenty days later, he was discharged home with his family tolerating a regular diet, his tracheostomy had been removed and was ambulating with minimal assistance.

## 3. Discussion

ECMO is an established salvage therapy for profound respiratory failure and has recently been shown to be superior to conventional ventilator management in such patients [1]. As experience with extracorporeal circulation grows, unique applications have been described. The need for systemic anticoagulation has often been contraindicated use in patients with severe intracranial pathology, and in particular, recent hemorrhage. However, advances in circuit and oxygenator technology, such as heparin bonding, have challenged this concern and cases of ECMO support with intracranial pathology have been recently described. Given our recent previous experiences with systemic anticoagulation in patients with significant intracranial pathology requiring life-saving ECMO [4] and cardiopulmonary bypass [5, 6], had we known about his intracranial bleed prior to cannulation, we probably would have altered our initial dose of heparin slightly (e.g., 5,000 or 7,500 unit bolus), but we still would have systemically anticoagulated this patient. While anticoagulation can often be held for several days once ECMO is initiated, as was done in this case, unfortunately some initial anticoagulation is necessary during the initial placement of cannulas to prevent them from clotting before extracorporeal blood flow can be started (as we have observed in previous clinical experiences in which patients were not anticoagulated during cannulation). While this initial bolus of heparin could worsen, or even precipitate, intracranial or body cavity bleeding, such decisions are potentially unavoidable and need to be balanced against clinical needs for a life-saving intervention. Clearly, the risks and benefits need to be weighed in such extreme situations such as this case in which definitive imaging was not obtainable due to patient physiologic instability prior to implementation of ECMO.

The management of patients presenting with profound hypothermia from environmental exposure is dependent upon the degree of hypothermia and the physiologic consequences. In mild cases, typically topical warming blankets, warming of intravenous fluids, and, if intubated, warming of inhaled oxygen are typically sufficient. However, in more severe cases, active core warming with extracorporeal circuits has been associated with a >6-fold increase in survival when the presenting core temperature is <30C [2].

In addition, the use of ECMO for respiratory failure following trauma, either as a consequence of primary causes such as pulmonary contusions, respiratory failure from fat embolism from long-bone fractures, and acquired pneumonias or secondary to systemic inflammatory syndromes such as adult respiratory distress syndromes has been described [7]. While survival rates of >50% have been reported in this extremely sick and complex patient population, typically these patients have already undergone extensive evaluation and preliminary management of their injuries. Furthermore, central nervous system trauma has been a contraindication to ECMO therapy [8]. Because CNS injuries contraindicate systemic anticoagulation, life-saving ECMO might not be offered in this patient population. Furthermore, delays in addressing profound hypoxemia while evaluating other potential injuries maybe contribute to secondary anoxic CNS injuries, potentially result in acute and lethal hemodynamic complications and additional end-organ failure.

The uniqueness of our case was the early implementation of ECMO as part of the initial trauma evaluation even before the secondary evaluation for other injuries could be completed, for the treatment of not only acute severe hypoxemia but also to assist in systemic rewarming for hypothermia. Although our patient was subsequently found to have a traumatic brain injury with intraventricular hemorrhage, we were able to maintain ECMO without anticoagulation for 48 hours at which time it was felt that his brain injury had stabilized to the point in which low-dose anticoagulation could be safely started. This management hypothesis was confirmed by lack of progression of his head injury on serial CT scans and stable intracranial pressures. Although venoarterial ECMO support would typically require full anticoagulation to prevent arterial embolization, an advantage of veno-veno support is the ability to manage the circuit with minimal or no anticoagulation, clearly an advantage in the polytrauma patient, particularly in those with a potential coagulopathy from both hypothermia and trauma [9]. While it can be argued that systemic heparinization might have precipitated or worsened the head bleed in our patient, we felt that given the nature of our patient's presentation and adhering to the basic “airway breathing circulation” principles of resuscitation that stabilizing his oxygenation and ventilation was our only option prior to proceeding with further evaluation and management.

## 4. Conclusions

Extracorporeal membrane oxygenation is an acceptable therapy for patients with profound respiratory failure and systemic hypothermia secondary to trauma. When indicated, therapy should be initiated immediately as a component of the initial trauma evaluation. Delays in therapy, while other injuries are defined and managed, may contribute to further anoxic insults and even death. Such interventions may be beneficial and should be considered even in patients with severe intracranial pathology such as traumatic intraventricular hemorrhage.
